# Hyperammonemic Coma in an Adult due to Ornithine Transcarbamylase Deficiency

**DOI:** 10.1155/2013/493216

**Published:** 2013-02-12

**Authors:** Daniel L. Roberts, David A. Galbreath, Bhavesh M. Patel, Timothy J. Ingall, Amer Khatib, Daniel J. Johnson

**Affiliations:** ^1^Division of Hospital Internal Medicine, Department of Medicine, Mayo Clinic, Phoenix, AZ 85054, USA; ^2^Kansas City Family Medical Clinic, Kansas City, MO 64114, USA; ^3^Department of Critical Care Medicine, Mayo Clinic, Phoenix, AZ 85054, USA; ^4^Department of Neurology, Mayo Clinic, Phoenix, AZ 85054, USA; ^5^Division of Gastroenterology, Faculty of Medicine, University of Jordan, Amman 11942, Jordan; ^6^Department of Surgery, Mayo Clinic, Phoenix, AZ 85054, USA

## Abstract

*Objective*. To report an unusual cause of coma in an adult. *Design*. Case report. *Setting*. University teaching hospital. *Patient*. A previously healthy 53-year-old man initially presented with altered mental status and progressed to coma. He was found to be substantially hyperammonemic and did not improve with lactulose therapy and continuous venovenous hemodialysis. *Results*. Biochemical testing revealed previously undiagnosed ornithine transcarbamylase deficiency, and the patient responded to arginine, sodium phenylacetate, and sodium benzoate. *Conclusion*. Even in adult patients with no known history, inborn errors of metabolism must be considered in the differential diagnosis of unexplained coma. Defects of the urea cycle can present with an unprovoked hyperammonemic coma.

## 1. Introduction

The differential diagnosis of coma in adults is notoriously broad, but the presence of hyperammonemia narrows it to Reye's syndrome, high-dose chemotherapy, *Proteus *infection, glycine toxicity (after transurethral resection of prostate) [1], liver failure, and late-onset urea cycle defects [2]. Although disorders of the urea cycle are predominantly the purview of the neonatologist, presentations of urea cycle enzyme deficiencies in adults have been reported.

## 2. Case Report

A fifty-three-year-old man was admitted to an outside hospital with a significantly decreased level of consciousness. He had awoken at night and urinated on the bathroom floor, and the following morning, his wife was unable to fully awaken him. He had reported progressive fatigue over the preceding two weeks, but had otherwise been in his usual state of good health. Specifically, he had no history of fevers, chills, rigors, weight loss, chest pain, shortness of breath, nausea, vomiting, or abdominal pain.

His past medical history was significant only for benign prostatic hypertrophy and hyperlipidemia. He had no history of alcohol abuse, diabetes mellitus, or liver disease. His medications included terazosin, tamsulosin, and a recent methylprednisolone taper for a nonspecific groin rash. He had initiated therapy with simvastatin approximately one month prior to admission. His only recent toxin exposure was the use of a permethrin-based insecticide, and he reported no symptoms during or after this exposure.

On admission to the outside hospital, the patient was afebrile and anicteric with normal vital signs. The neurologic examination revealed that he was only intermittently arousable to verbal stimuli, with the remainder of the exam being nonfocal. A complete blood count, serum electrolytes, serum alcohol, cardiac enzymes, urine drug screen, chest X-ray, CT scan of the head, and MRI/MRA of the brain were normal. His ammonia level was noted to be elevated at 202 umol/L (normal range = 11–35 umol/L). His other liver function tests were as follows: AST = 19 units/L (8–14 units/L), ALT = 70 units/L (7–55 units/L), total bilirubin = 1.4 mg/dL (0.1–1.0 mg/dL), and INR = 1.12. A CT scan of the abdomen revealed fatty infiltration of the hepatic parenchyma, but was otherwise unremarkable. Over the course of his stay, he became less responsive, and his ammonia level failed to normalize despite aggressive lactulose therapy. He was intubated for airway protection and transferred to our institution for further evaluation.

On arrival, the patient withdrew to pain in his lower extremities, with no response in the upper extremities. Pupils were equal and reactive, deep tendon reflexes were normal and symmetric, and his left plantar response was equivocal. His Glasgow Coma Scale score was 10 (E4, V2, M4). His vital signs and the remainder of his physical examination were unremarkable. Initial laboratory data were significant for a leukocytosis (16.6 × 10^9^/L, normal range 3.5–10.5 × 10^9^/L), hypernatremia (155 mEq/L, 135–145 mEq/L), hypocalcemia (8.8 mg/dL, 8.9–10.1 mg/dL), an AST level of 34 units/L, an ALT level of 50 units/L, an ammonia level of 215 ug/dL, and an INR of 1.21. Alkaline phosphatase and total bilirubin were within normal limits. MRI of his brain and cervical spine was negative and specifically revealed no evidence of spinal cord compression as a cause for his upper extremity weakness. An electroencephalogram (EEG) showed diffuse slowing and triphasic waves consistent with hepatic encephalopathy. Quantitative serum amino acids, urine organic acid screen, and urine heavy metal screen were drawn, while lactulose and respiratory support were continued.

**Figure 1:**
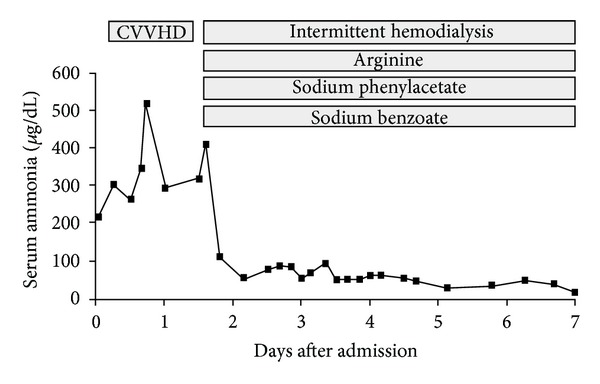
The patient's serum ammonia charted against time after admission, with graphic representation of timing of interventions. CVVHD: continuous venovenous hemodialysis.

**Figure 2:**
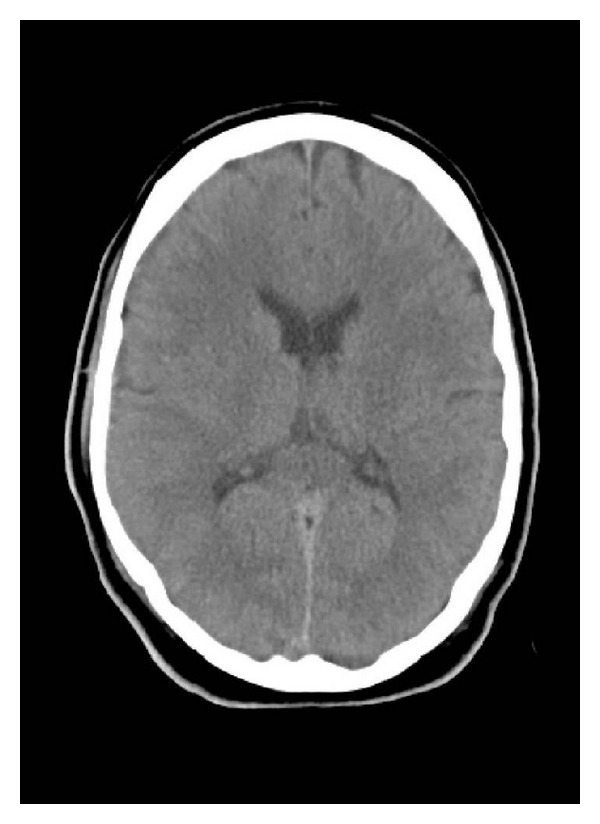
CT of the head without contrast reveals evidence of mild cerebral edema.

**Figure 3:**
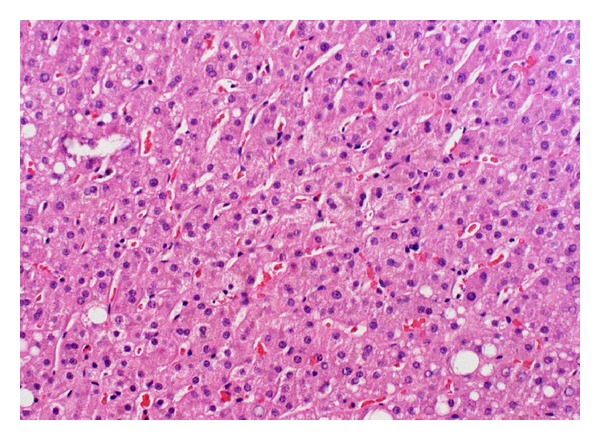
Liver biopsy reveals centrilobular steatosis without fibrosis. (Hematoxylin and eosin, 40x.)

 Over the next twelve hours, the patient's ammonia level worsened to 516 ug/dL (Figure 1). Moderate flux continuous venovenous hemodialysis (CVVHD) was initiated, and his ammonia decreased to 291 ug/dL without any observable improvement in his level of consciousness. A repeat CT of his head demonstrated new cerebral edema (Figure 2). A subarachnoid bolt was placed and revealed normal intracerebral pressures. Transcutaneous liver biopsy was obtained, revealing mild-to-moderate centrilobular steatosis without evidence of fibrosis (Figure 3). This was felt to be inconsistent with the diagnosis of Reye's syndrome.

At this time, laboratory data returned revealing a marked elevation in the patient's urine orotic acid and uracil and confirming the diagnosis of ornithine transcarbamylase deficiency. The patient was subsequently treated with an infusion of intravenous arginine with dextrose. On hospital day 2, his ammonia level worsened to 410 ug/dL while on CVVHD, and there was a deterioration of lower extremity withdrawal strength to painful stimuli. He was switched to intermittent hemodialysis. He was also started on intravenous sodium phenylacetate and sodium benzoate. His ammonia level subsequently normalized, and his mental status improved to the point that he was extubated on hospital day 7. When he resumed oral intake, he was started on oral sodium phenylacetate, sodium benzoate, and a protein-restricted diet. His mental status subsequently returned to baseline, and he was discharged home. His simvastatin was restarted by his primary physician with no adverse effects. The patient's family initially denied any family history of metabolic disorders. However, after some investigations, they subsequently remembered that the patient's maternal second cousin had been diagnosed with OTC deficiency. His relatives were tested for OTC deficiency. The patient currently takes arginine and sodium phenylbutyrate and has not suffered any further complications of his disease over eleven years of followup.

## 3. Discussion

**Figure 4:**
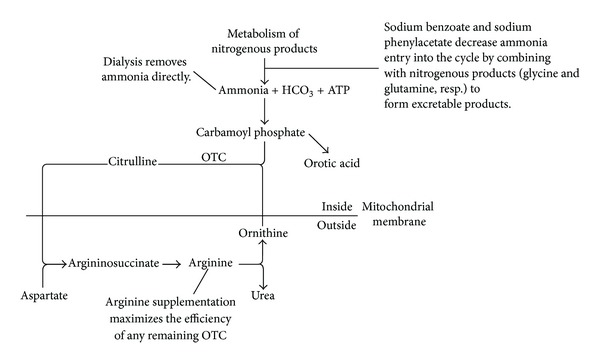
The urea cycle, demonstrating steps at which interventions are targeted in OTCD. ATP: adenosine triphosphate. ASAS: argininosuccinate synthetase. ASAL: argininosuccinate lyase. ARG-1: arginase 1.

 The urea cycle is the primary metabolic pathway for the excretion of nitrogenous wastes as urea. Ornithine transcarbamylase (OTC) catalyzes the second of the five reactions that make up the cycle (Figure 4). It catalyzes the mitochondrial reaction of ornithine (the end product of the extraction of urea from arginine) with carbamoyl phosphate (the first storage form of ammonia) to produce citrulline. Deficiency of OTC leads to the formation of excess carbamoyl phosphate, some of which is excreted as an orotic acid. When this pathway is overwhelmed, hyperammonemia results. Thus, patients with OTCD tend to have hyperammonemia, elevated levels of orotic acid in the urine, and low plasma citrulline. 

OTCD is the most common urea cycle defect, with an estimated incidence of one in 14,000 live births [3]. The OTC gene is located on the short arm of the X chromosome, and its X-linked recessive inheritance makes it the only urea cycle defect not to be inherited in an autosomal recessive manner [4]. Most males with the deficiency die as neonates. However, phenotypic variability has been noted, and cases of patients presenting as adults have been reported [5, 6]. Female heterozygotes are also occasionally noted to manifest symptoms [7, 8].

 Symptoms of late-onset OTCD are those of hyperammonemia, and include lethargy, confusion, nausea, vomiting, bizarre behavior, coma, and death. During more severe attacks, intracranial edema and a resulting rapidly fatal course are known complications [9]. Animal models have suggested that this edema may be due to selective astrocytic swelling secondary to hyperammonemia [2].

 Hyperammonemia associated with OTCD requires prompt identification and treatment to avoid adverse neurological sequelae and death. Multiple modalities for lowering the serum ammonia level are generally applied simultaneously. On an emergent basis, ammonia can be filtered from the blood via hemodialysis or continuous venovenous hemodiffusion. Lactulose is given simultaneously in a manner similar to that used in hyperammonemia secondary to hepatic failure. Finally, a dextrose infusion is started to provide nonprotein caloric intake and prevent proteolysis.

While these immediate interventions are underway, intravenous sodium benzoate and sodium phenylacetate are given to shunt nitrogen-containing compounds away from the urea cycle [10]. Sodium benzoate conjugates with glycine to form hippuric acid, and sodium phenylacetate conjugates with glutamine to form phenylacetylglutamine. Both end products are readily excreted in the urine. Since adults presenting with OTCD are assumed to have only partial deficiencies, arginine has been given to provide ornithine substrate to react with the resulting excess carbamoyl phosphate and, as a result, to maximize activity of the remaining OTC enzyme. Because patients with OTCD are generally arginine deficient, its replenishment also tends to decrease proteolysis, thus diminishing traffic through the urea cycle. Finally, pyridoxine and folate can be supplemented to drive glycine synthesis and maximize the effectiveness of sodium benzoate [11].

The increased effectiveness of HD over CVVHD in the setting of hyperammonemia due to OTCD (as manifested by this patient) has been previously reported [12].

 The specific trigger for this patient's presentation with life-threatening hyperammonemia remains unclear. Typical triggers (increased protein ingestion, infection, perioperative or postpartum state, and anticonvulsant medications) were not noted. No prior reports could be found linking the use of simvastatin or any other HMG Co-A reductase inhibitor with the unmasking of OTCD. Immunosuppressant doses of corticosteroids have been found to trigger attacks of hyperammonemia in OTCD [13], but not at the doses our patient briefly received for his nonspecific dermatitis.

Also poorly understood is the degree and pattern of weakness that he manifested. Although an imaging study suggested mild cerebral edema, invasive intracranial pressure monitoring did not show any evidence of elevated pressures. His neurological recovery was complete.

In summary, late-onset OTCD is uncommon, but it must be considered in the differential diagnosis of hyperammonemia in the setting of relatively normal hepatic enzymes and synthetic function. Although it is frequently fatal, prompt diagnosis and initiation of definitive therapy can be lifesaving.
